# The Effects of Iron Rust on the Ageing of Woods and Their Derived Pulp Paper

**DOI:** 10.3390/polym13203483

**Published:** 2021-10-11

**Authors:** Wael A. A. Abo Elgat, Ayman S. Taha, Mohamed Z. M. Salem, Yahia G. D. Fares, Martin Böhm, Mourad F. Mohamed, Ramadan A. Nasser, Vojtěch Pommer

**Affiliations:** 1 Restoration Department, High Institute of Tourism, Hotel Management and Restoration, Abukir, Alexandria 21526, Egypt; watsat20@yahoo.com; 2 Conservation Department, Faculty of Archaeology, Aswan University, Aswan 81528, Egypt; aymansalahtaha82@yahoo.com; 3 Forestry and Wood Technology Department, Faculty of Agriculture (EL-Shatby), Alexandria University, Alexandria 21545, Egypt; nasser67@alexu.edu.eg; 4 General Laboratory and Research, Misr Edfu Pulp Writing and Printing Paper Co. (MEPPCO), Aswan 81656, Egypt; yahyagml@yahoo.com; 5 Department of Materials Engineering and Chemistry, Faculty of Civil Engineering, Czech Technical University in Prague, Thákurova 7, 166 29 Prague 6, Czech Republic; martin.bohm@fsv.cvut.cz (M.B.); vojtech.pommer@fsv.cvut.cz (V.P.); 6 Restoration Department, Faculty of Archaeology, Cairo University, Giza 12613, Egypt; msms147@yahoo.com

**Keywords:** iron rust, mechanical properties, wood pulp, Fourier transform infrared spectroscopy, accelerated ageing

## Abstract

The accelerated ageing of wood in terms of heating or iron rusting has a potential effect on the physio-mechanical, chemical and biological properties of wood. The effects of accelerated ageing on the mechanical, physical and fungal activity properties of some wood materials (*Schinus terebinthifolius*, *Erythrina humeana*, *Tectona grandis*, *Pinus rigida* and *Juglans nigra*) were studied after several cycles of heating and iron rusting. The fungal activity was assayed against the growth of *Aspergillus terreus*, *Aspergillus niger*, *Fusarium culmorum* and *Stemphylium solani*. In addition, the mechanical and optical properties of paper sheets produced from those wood pulps by means of Kraft cooking were evaluated. The mechanical and chemical properties of the studied wood species were affected significantly (*p* < 0.05) by the accelerated ageing, compared to control woods. With Fourier transform infrared (FTIR) spectroscopy, we detected an increase in the intensity of the spectra of the functional groups of cellulose in the heated samples, which indicates an increase in cellulose content and decrease in lignin content, compared to other chemical compounds. For pulp properties, woods treated by heating showed a decrease in the pulp yield. The highest significant values of tensile strength were observed in pulp paper produced from untreated, heated and iron-rusted *P. rigida* wood and they were 69.66, 65.66 and 68.33 N·m/g, respectively; we calculated the tear resistance from pulp paper of untreated *P. rigida* (8.68 mN·m^2^/g) and *T. grandis* (7.83 mN·m^2^/g) and rusted *P. rigida* (7.56 mN·m^2^/g) wood; we obtained the values of the burst strength of the pulp paper of untreated woods of *P. rigida* (8.19 kPa·m^2^/g) and *T. grandis* (7.49 kPa·m^2^/g), as well as the fold number of the pulp paper of untreated, heated and rusted woods from *P. rigida*, with values of 195.66, 186.33 and 185.66, respectively. After 14 days from the incubation, no fungal inhibition zones were observed. Accelerated ageing (heated or iron-rusted) produced significant effects on the mechanical and chemical properties of the studied wood species and affected the properties of the produced pulp paper.

## 1. Introduction

Wood, as a natural material, can be deteriorated by biodegradation, weathering and ageing. Weathering (the slow degradation of materials exposed to the weather) and accelerated weathering (the laboratory emulation of the damaging factors of weather for the aim of anticipating the relative strength of materials exposed to outdoor environments) have been studied for their effects on the properties of wood [1,2,3,4,5,6,7,8]. By investigating the heat treatment, the ageing mechanism of wood can be evaluated [9,10]. Heat, under dry circumstances, is assumed to be accompanied by thermal oxidation of the wood ingredients, whereas, under wet conditions, the participation of hydrolysis can be more frequently expected [11,12,13]. A lower equilibrium of the moisture content was found as a result from thermally treating wood [14].

A weakening in wood can be observed by the corrosion of metal linked with wood in different shapes, such as spikes, bolts, boat docks, nails, screws, plates, rail ties and wooden vessels [15,16]. The degradation of the wood around the fastener is caused by the severely corroding metal corrosion products, when preservative or fire treatments are utilized [17], as well as by the occurrence of H_2_O and O_2_ in the cellular structure of wood [18].

Wet wood not only causes metals to corrode, but certain conditions are created when a metal is embedded in wet wood, which can accelerate the corrosion of the metal [19]. Strength loss occurs as a result of the corrosion of the metal with the deterioration of the wood. The wooden parts surrounding the nails are usually stained black and degraded because of the acid that accumulates in the crevice and wood fibers [15,20].

Furthermore, the corrosion of metals embedded in wood are affected by three groups of extractives organic acids (found in black liquors of wood pulp), tannins and phenols [21]. Fungal pathogens require from 10^−7^ to 10^−6^ M iron for their growth [22,23] and iron in the status of Fe(II) and Fe(III) is required by most living systems [24]. Iron is used as a cofactor for oxidation-reduction enzymes and it is essential for fungal metabolism to prevent repolymerization and toxicity [25]. In the form of phenolate, iron is produced by the wood-rotting fungus *Gloeophyllum trabeum* that conjugates with cellulose and activates the degradation mechanism [26]. On the other hand, excess iron is toxic, as it mediates the formation of potentially deleterious free radicals, and the inactivation of iron assimilation mechanisms attenuates the virulence of major fungal pathogens such as *Candida albicans* and *Cryptococcus neoformans* [27].

Iron is required by most living systems, since it has two readily available ionization states, Fe(II) and Fe(III) [28], and it is an essential micronutrient for most organisms. Fungal pathogens require from 10^−7^ to 10^−6^ M iron for their growth [22]. On the other hand, excess iron is toxic, as it mediates the formation of potentially deleterious free radicals [27]. Iron is toxic in uncontained situations because it catalyzes the production of free radicals. Therefore, after uptake, storage of the accessed iron becomes essential in the fungal metabolism of the metal to prevent repolymerization and toxicity [28].

In the last years of the 19th century, with the increasing demand for paper and the scarce availability of cellulose fibers from linen, agricultural residues and cotton [29,30,31], wood became the main source of this compound. It is evident that the evolution in the manufacture of paper through the centuries clearly shows a marked modification in its structure, both in relation to the origin of cellulose fibers [32] and to the types of additives used. In this context, the process of sizing plays a key role, directly affecting the mechanism of water and ink absorption by paper fibers [33]. Iron ions, as impurities, can catalyze the oxidation processes in cellulose, probably as centers able to form free radicals through a sequence of Fenton reactions [34]. Paper products are always faced with physical damages, such as yellowing and pollution problems [35], because of the low glass transition temperature (T_g_). Regarding the physical characteristics, the most proper material for the restoration of paper products should always necessarily meet two demands, including legibility (transparency) and durability against ageing [36].

Wood, wood-derived paper sheets and iron are indispensable materials widely used in construction, furniture and various tools. The combined use of these materials is necessary for many purposes and, as such, the contact between iron and wood/paper sheets is unavoidable. Under natural and room conditions, iron can rust and the formed rust likely promotes the ageing of the wood and paper sheet with which it comes into contact, resulting in changes in their physical, chemical and biological properties. To provide a detailed understanding of these potential effects, this study was carried out. This is an issue that needs to be studied because the rusting process of iron adjacent to the wood can negatively affect system performance. Therefore, this work aims at evaluating the effect of heating and iron rusting on the physical, mechanical, chemical and biological properties of five wood species subjected to accelerated ageing. Additionally, the produced pulp paper from those woods were evaluated in terms of their mechanical and optical properties.

## 2. Materials and Methods

### 2.1. Preparation of Raw Materials

Samples of stem wood were used from two local tree species, *Schinus terebinthifolius* and *Erythrina humeana*, beside three imported woods, *Tectona grandis*, *Pinus rigida* and *Juglans nigra*. All wood samples were air-dried at laboratory conditions (temperature of 27 ± 3 °C and relative humidity (RH) of 65 ± 5%). Wood samples were prepared with different dimensions, namely, 2.5 cm × 2.5 cm × 10 cm, 30 cm × 2 cm × 2 cm and 30 cm × 2 cm × 0.6 cm. Samples from each wood were divided into three groups: samples from the first group were left in their air-drying status as control treatment; the second group was subjected to accelerated ageing in water (heated); the third group was subjected to accelerated ageing in water suspended with iron rust. There were three replicates for each mechanical characteristic studied. Three wood samples from each wood species were used for each treatment.

### 2.2. Ageing Processes

Several studies have been conducted to measure the accelerated exposure test that consists in the exposure of samples to certain conditions of temperature and humidity, based on the standard methods and from the published literature [11,17,37,38,39,40,41,42,43,44]. In order to obtain a realistic simulation of the wooden elements in contact with iron in the surrounding environment, accelerated artificial ageing stages were carried out on the wood samples of the second group through four successive cycles. One cycle consisted of four steps, as shown in Table 1 [45,46].

The four stages in Table 1 represent one ageing cycle; this cycle was repeated four consecutive times for the second group of samples (heated woods). On the other hand, the third group of samples was subjected to the same above-mentioned stages, but with water being replaced by water suspended with iron rust (100 g/L of water) (iron-rusted woods). This means that the iron-rust solution used in the ageing process for the third group of samples was prepared by adding 100 g per liter (10%) of water and stirring to obtain the iron rust suspension. This method somewhat simulates the case of wood coming into contact with iron and being exposed to high levels of relative humidity in the surrounding atmosphere, or immersed in water or in moist soil. 

### 2.3. Mechanical, Physical, Chemical and Biological Properties

#### 2.3.1. Mechanical and Physical Properties

After sample conditioning [47], the tested wood samples were measured for their mechanical properties according to the British standard specification [48]. Three mechanical tests were carried out, namely, compression strength (*C_max_*) parallel to grain (MPa), modulus of rupture (MOR) in bending (MPa) and maximum tensile strength (MTS) parallel to grain (MPa). All tests were carried out using the Quasar 600 kN instrument (Galdabini Spa, Cardano al Campo, Italy). The static quality value was calculated as a ratio of the crushing strength parallel to the grain and 100× air-dry density of wood [49,50,51].

The wood species were prepared in different dimensions: 2.5 cm × 2.5 cm × 10 cm to measure the compression parallel to grain [52]; 30 cm × 2 cm × 2 cm to test static bending strength [48]; 30 cm × 2 cm × 0.6 cm to test the tensile strength parallel to grain [48].

The density of wood and moisture content (MC%) were determined after the bending-strength test by cutting off at least three pieces with the dimension 2 cm × 2 cm × l.5 cm near the failure region [53]. Based on the oven-dry weight (o.d.) and volume at the time of testing, the density of each specimen was calculated using the dimension method [54], while the MC was determined based on the o.d. weight [55].

The mechanical and physical properties of the age-accelerated groups (heated and iron-rusted) were compared with the control group (without accelerated ageing).

#### 2.3.2. Chemical Composition of Wood Species

All wood samples (untreated, heated and iron-rusted) were chipped, fractionated using a knife mill and screened; the size of the chips was set as 20 mm long and 13 mm wide. The samples from homogenized wood chips from *S. terebinthifolius*, *E. humeana*, *T. grandis*, *P. rigida* and *J. nigra* were milled and sieved and the 40–60 mesh fraction was used for the summative chemical analysis. The extractive contents (alcohol and benzene) were obtained using extraction thimbles in a Soxhlet apparatus for no less than 16 h according to the TAPPI standard method (T204). The thimbles were oven-dried and weighted after each extraction determining the extractive content by weight variation. Holocellulose was determined by using the modified chlorite TAPPI standard method T249; the insoluble lignin content was determined according to the TAPPI standard method (T222 om88). Ash content was determined by the TAPPI standard method (T211). All summative chemical analyses were reported as percentages of the initial mass.

#### 2.3.3. FTIR Spectroscopic Analysis of Accelerated Ageing of Wood Samples

The wood samples were analyzed with a Nicolet 380 FT-IR Spectrometer (Madison, WI, USA) using the solid-sample potassium bromide technique, at the National Institute for Measurement and Calibration, Tersa, Giza, Egypt, using the KBr pellet method at a resolution of 4 cm^−1^ ranging from 400 to 4000 cm^−1^. Standard Ø13 mm diameter pellets were prepared by mixing and pressing 10 mg of the dried wood extractive sample in 300 mg of KBr for 5 min under a pressure of 200 bar. Three parallel measurements were performed. The obtained FTIR spectra were further processed using the computer software Spectrum One (ver. 5.0.1) [56,57,58].

#### 2.3.4. Biological Activity of Accelerated Ageing of Five Wood Species In Vitro

**Figure 1:**
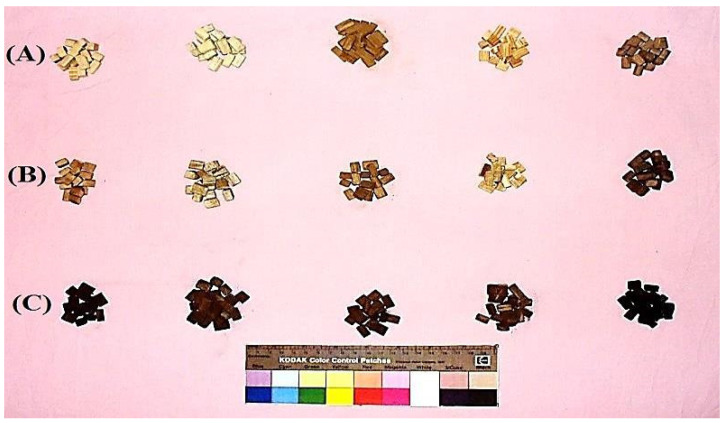
Groups of five age-accelerated wood species: (**A**) control, (**B**) heated and (**C**) rusted.

Wood blocks of 15 mm × 10 mm × 5 mm from each wood type were autoclaved at 121 °C for 20 min and left to cool. Figure 1 shows two groups (heated and iron-rusted) of five age-accelerated wood species and the control that were studied for their antifungal activity against four molds, namely, *Aspergillus terreus* Ate456, *A. niger* Ani245, *Fusarium culmorum* Fcu761 and *Stemphylium solani* Ssol382. A 14-day-old PDA culture of each fungus was prepared. After ageing, the groups of wood samples were inoculated with a disc (5 mm in diameter) of each fungus in a Petri dish that contained 15 mL of PDA culture and were incubated for one and two weeks at 25 ± 1 °C. Three replicates were used for each type of ageing samples. Five samples without ageing were used as control samples. Visual observation after 7 and 14 days of each group of wood samples against each fungus was performed and recorded using the recommendations of previously published works [59,60,61,62,63].

### 2.4. Pulp Production

#### 2.4.1. Kraft Pulping

Two hundred grams of o.d. wood chips from each wood species was swelled for one day, filtrated and impregnated in a sodium hydroxide 8% solution for 1 h at 85 °C; then, it was filtrated and washed from residual alkali with hot water at 70 °C.

Kraft pulping was conducted in a stainless-steel vessel with a capacity of 3 L under rotation in an oil bath. The cooking of wood chips (200 g based on o.d. weight) was carried out in two distinct stages. The first stage was a pretreatment, where wood chips were impregnated in a sodium hydroxide 3% solution for 4 h at 85 °C, then washed with hot water at 70 °C. The second stage is referred to as the post kraft cooking and was conducted for an additional 2.5 h. The wood pulping conditions were as follows, for all samples: active alkalinity charge, 18%; sulfidity, as sodium oxide, 20%; 175 °C cooking temperature; liquor ratio (liquid-to-wood chips ratio), 7:1. Subsequently, at the end of the cooking process, the rest of the chip pulp was disintegrated with a standard pulp disintegrator in 2 L of water for 15 min (about 50,000 revolutions). The solid residue was defibrated, washed with hot and cold water till neutral pH was reached; the resulting pulp was screened in a Valley flat screen having 0.25 mm slots and beat (Valley beater method), according to the TAPPI standard method T200 sp-96. All the wood chips were pulped in triplicate. Yield, Kappa number and freeness of pulp (Canadian standard method) were determined according to the TAPPI standard methods T210 cm-93, T236 om-13 and T227 om-99, respectively.

#### 2.4.2. Sheet Formation and Paper Testing

**Figure 2:**
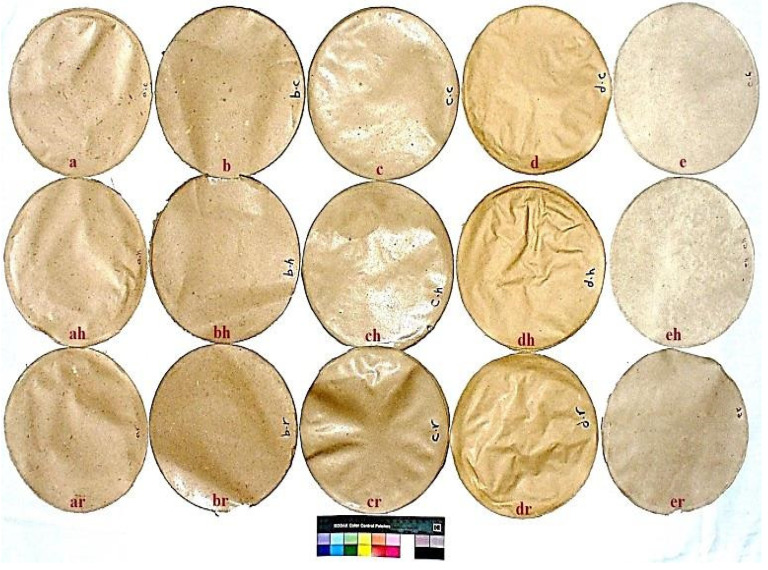
Paper sheets produced from wood species and after heating and iron rusting treatments; *Schinus terebinthifolius* (**a**), *Erythrina humeana* (**b**), *Tectona grandis* (**c**), *Pinus rigida* (**d**) and *Juglans nigra* (**e**). h, heated; r, iron-rusted.

The wood pulp was made into standard handsheets samples (200 cm^2^) with a grammage of about 60 g/m^2^ (TAPPI Standard T 205 sp-02) for determination of dry strength properties; the samples were conditioned at 50 ± 2% relative humidity and 23 ± 1 °C temperature according to TAPPI T 402 sp-98 for at least 4 h. Paper sheets (Figure 2) were made and tested for strength properties according to the TAPPI test methods T218 and T220. The handsheets were tested for tensile resistance (T403), tear strength (T414), bursting strength (T405), double fold number (T423) and optical properties (T452 om-92). All testing was in accordance with TAPPI standard test methods.

### 2.5. Statistical Analyses

The data were statistically analyzed with a two-way analysis of variance (ANOVA) using the SAS software [64], where the two factors were wood species and wood treatments (control, heated and iron-rusted). The comparisons among the treatments was measured using LSD 0.05.

## 3. Results

### 3.1. Properties of Age-Accelerated Wood

#### 3.1.1. Mechanical and Physical Properties of Age-Accelerated Woods

**Figure 3:**
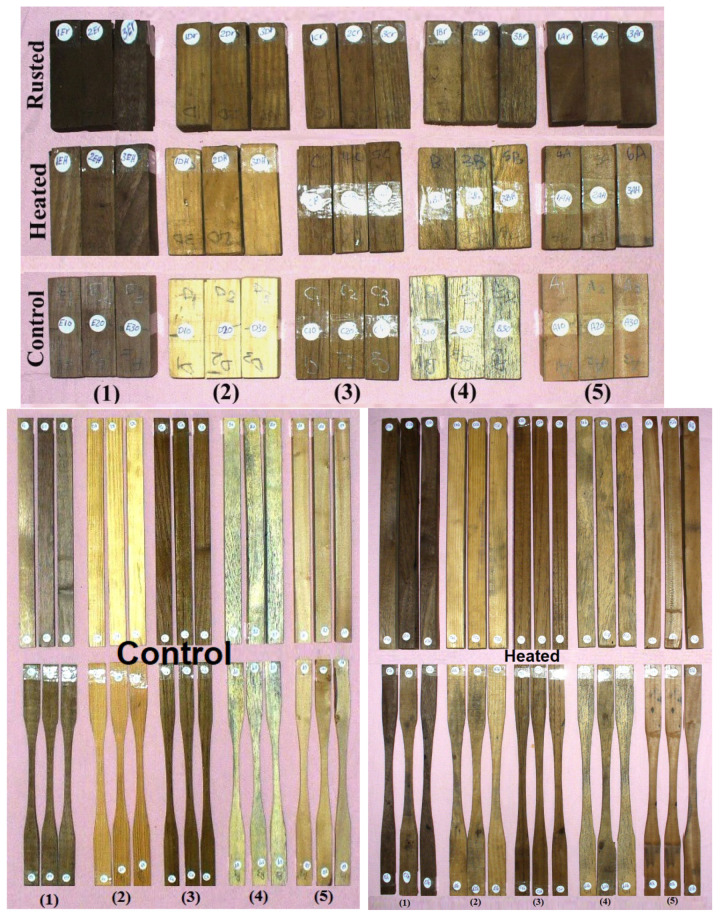

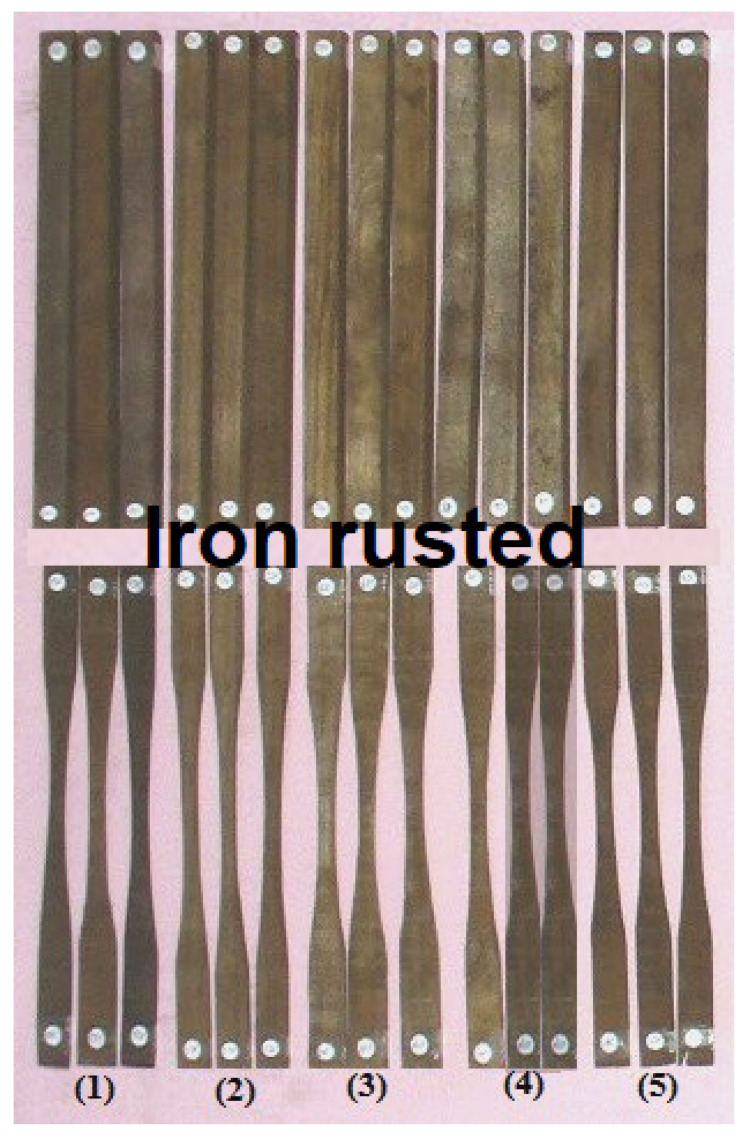
Wood samples with different treatments (heated and rusted woods) compared to control treatment (untreated wood) prepared for the testing of their mechanical properties. (1) *Schinus terebinthifolius*, (2) *Pinus rigida*, (3) *Tectona grandis*, (4) *Erythrina humeana* and (5) *Juglans nigra*.

Table 2 and Figure 3 present the values of selected mechanical and physical properties of the studied wood species subjected to different treatments (heated and iron-rusted), compared to control treatment (untreated wood). It can be seen that the highest MOR values were 144.18 and 139.65 MPa, obtained from rusted *J. nigra* and heated *P. rigida* wood, respectively, followed by untreated *T. grandis* wood (129.18 MPa), untreated *J. nigra* wood (126.10 MPa), rusted *P. rigida* wood (124.87 MPa) and *T.* rusted *grandis* wood (123.70 MPa). The lowest values observed were obtained from untreated, heated and rusted *E. humeana* wood with values of 28.91, 28.03 and 23.53 MPa, respectively. The highest values of maximum tensile strengths (MTS) were observed in the untreated wood of *P. rigida* (130.52 MPa), *T. grandis* (109.85 MPa) and *J. nigra* (103.17 MPa) and in heated *T. grandis* wood (102.32 MPa). Untreated, heated and rusted *E. humeana* wood showed the lowest MTS values—20.74, 16.29 and 16.38 MPa, respectively. The maximum crushing strength (C_max_) values were reported for untreated, heated and rusted *T. grandis* wood, with values of 65.26, 64.46, 58.75 MPa, respectively, while the lowest values were observed in tested untreated, heated and rusted *E. humeana* wood, with values of 11.94, 12.12 and 13.54 MPa, respectively. Other treated woods showed values ranging from 43.40 to 57.82 MPa.

As for the density of the tested woods, the statistical model used showed no significant differences among the samples, but the highest density values were found in all wood species within their treatments (0.538–0.599 g/cm^3^) except for the values obtained from *E. humeana* wood (0.214–0.227 g/cm^3^). The moisture content for all the tested wood species and their treatments was in the range of 7.34–12.51%, with no significant differences among the samples.

#### 3.1.2. Chemical Composition of the Raw Materials

Table 3 shows the chemical analysis of the studied wood species as affected by heating or iron-rusted, compared with untreated woods (control). The highest contents (%) of the alcohol and benzene extractives were recorded in the untreated woods from *S. terebinthifolius*, *T. grandis* and *E. humeana*, with percentages of 9.30, 8.23 and 8.16%, respectively, while the rusted, heated and control woods of *P. rigida* showed the lowest amounts, with percentages of 2.56, 3.33 and 4.36%, respectively. The lignin content (%) was found in high percentages in the untreated, heated and rusted wood from *T. grandis,* with values of 29.33, 29.66 and 29.66%, respectively, and in rusted *P. rigida* (29.60%), while the lowest amounts were observed in the untreated, heated and rusted wood of *J. nigra* with values of 22, 22.33 and 22.60, respectively. *J. nigra* wood showed the highest content of holocellulose, with 69.97% (untreated), 71.25% (heated) and 71.46% (rusted), while *T. grandis* wood showed the lowest content of holocellulose, with 59.95% (untreated), 61.22% (heated) and 61.34% (rusted). Untreated and heated wood of *E. humeana* showed the highest amount of ash content, with percentages of 3.96 and 3.40%, respectively, followed by untreated woods from *T. grandis* (2.86%), *S. terebinthifolius* (2.86%) and *E. humeana* (2.37%); *P. rigida* wood showed the lowest ash content among the studied wood samples with percentages of 0.85% (heated) and 0.43% (rusted), while from rusted *J. nigra* wood we obtained a value of 0.62l%.

#### 3.1.3. FTIR Analyses of Accelerated Ageing of Wood Samples

**Figure 4:**
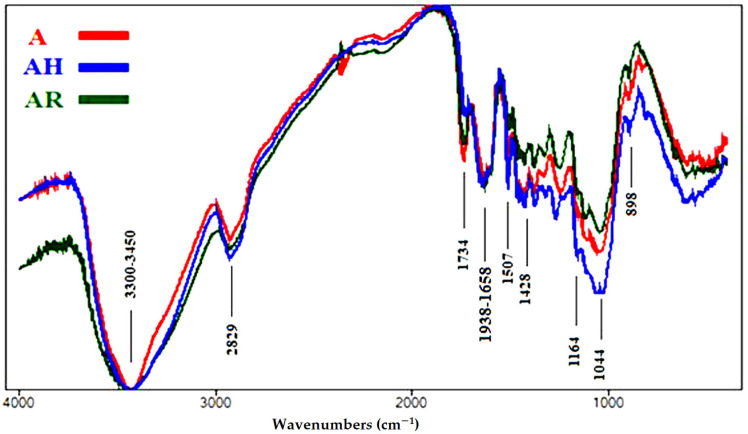
FTIR spectra of the wood samples of *Schinus terebinthifolius* (A, control; AH, heated ageing; AR, rusted ageing).

**Figure 5:**
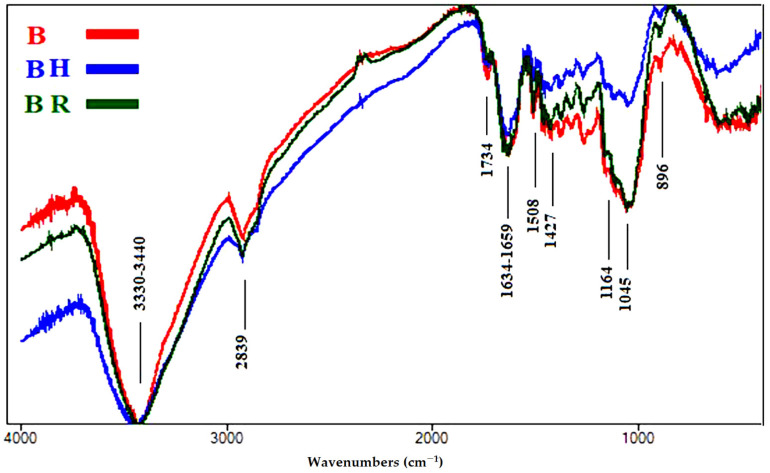
FTIR spectra of the wood samples of *Erythrina humeana* (B, control; BH, heated ageing; BR, rusted ageing).

**Figure 6:**
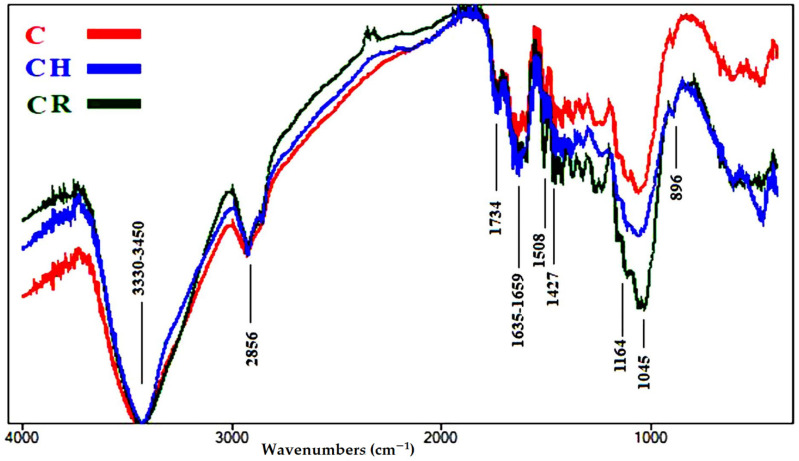
FTIR spectra of the wood sample of *Tectona grandis* (C, control; CH, heat ageing; CR, rusted ageing).

**Figure 7:**
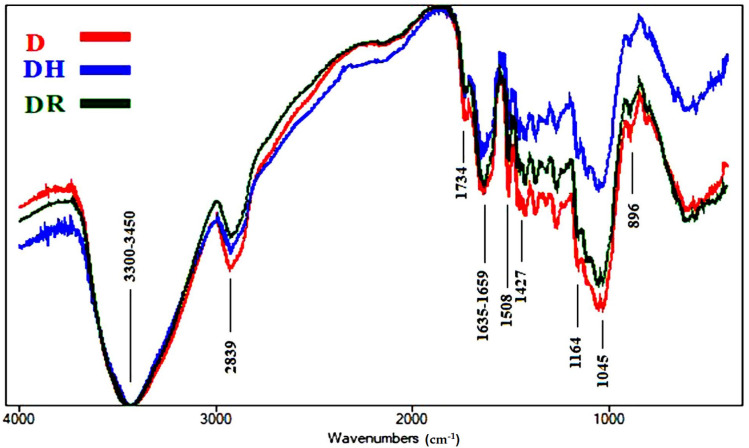
FTIR spectra of the wood samples of *Pinus rigida* (D, control; DH, heated ageing; DR, rusted ageing).

**Figure 8:**
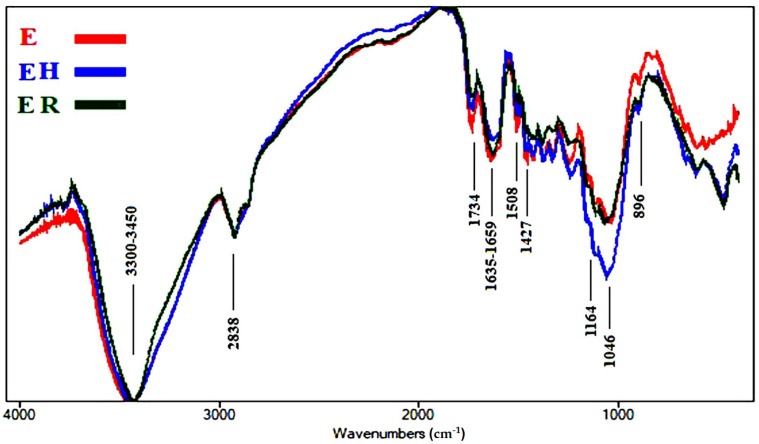
FTIR spectra of the wood sample of *Juglans nigra* (E, control; EH, heated ageing; ER, rusted ageing).

Fourier transform infrared (FTIR) spectroscopy is considered an established technique used to determine the chemical composition of various chemical samples [57,65,66,67]. The intensities of the functional chemical groups are reported in Table 4. The FTIR spectra of the aged wood samples are presented in Figure 4, Figure 5, Figure 6, Figure 7 and Figure 8. 

Current FTIR spectroscopic studies mostly deal with the structure of wood after diverse modifications. Chemical changes in the molecular structure of wood exposed to natural or artificial weathering have been monitored with various FTIR techniques; besides, IR spectroscopy was exercised to reveal thermal modifications in wood and lignin [68].

Figure 4 shows the FTIR spectra of the wood sample from *S. terebinthifolius*, where a few changes occurred. The O-H stretching intensity at 3300–3450 cm^−1^ was almost negligible in all three samples treated; we observed a slight decrease in unconjugated C=O stretching broadening at 1734 cm^−1^ for hemicellulose in the rusted sample (AR) and a decrease in it in the heated sample (AH). No changes occurred in conjugated C=O stretching broadening at 1638–1658 cm^−1^, which expressed the oxidation of cellulose, in none of the samples. No changes occurred in C=C stretching broadening at 1507 cm^−1^, related to lignin, in AH, compared with the control (A), while there was a clear decrease in intensity in AR. No changes occurred in CH_2_ bending at 1428 cm^−1^, related to cellulose (crystallized and amorphous), in none of the samples. A slight decrease in O-C-O stretching broadening at 1164 cm^−1^, which expressed the polymerization of cellulose, was noted in AR, while there was a clear increase in intensity in AH. No changes occurred in O-C-O stretching at 898 cm^−1^, related to crystallized cellulose, in none of the samples. The decrease in lignin in AH indicates an expected decrease in mechanical compressive strength.

Figure 5 shows the FTIR spectra of the wood samples of *E. humeana*, where slight changes occurred. A slight increase in O-H stretching broadening at 3330–3440 cm^−1^ in the rusted sample (BR) was found, while there was a clear increase in broadening in the heated sample (BH). A slight decrease in unconjugated C=O stretching broadening at 1734 cm^−1^, which expressed hemicellulose, was noted in all samples. No changes occurred in conjugated C=O stretching broadening at 1635–1659 cm^−1^, related to cellulose oxidation, in BR, while there was a slight decrease in intensity in BH. No changes occurred in C=C stretching broadening at 1508 cm^−1^, related to lignin, in BR, compared with the control (B), while there was a clear decrease in intensity in BH. No changes occurred in CH_2_ bending at 1427 cm^−1^, which expressed cellulose (crystallized and amorphous), in BR, compared with B, while there was a clear decrease in intensity in BH. A slight decrease in O-C-O stretching broadening at 1164 cm^−1^, expressing cellulose polymerization, was recorded in BR, while there was a clear decrease in intensity in BH. No changes occurred in O-C-O stretching at 896 cm^−1^, which expressed crystallized cellulose, in none of the samples. The decrease in lignin in BH indicates an expected decrease in mechanical compressive strength. Increasing the water content in the wood samples affected the static bending strength in the treated wood samples, especially in BR.

Figure 6 shows the FTIR spectra of the wood sample of *T. grandis*, where a few changes occurred. The O-H stretching intensity at 3300–3450 cm^−1^ was almost negligible in all three samples. No changes occurred in unconjugated C=O stretching broadening at 1734 cm^−1^, related to hemicellulose, in none of the samples. A slight increase was observed in conjugated C=O stretching broadening at 1635–1659 cm^−1^, for cellulose oxidation, in the rusted sample (CR) and in the heated sample (CH), compared with the control (C). No changes occurred in C=C stretching broadening at 1508 cm^−1^, for lignin, in none of the samples. No changes occurred in CH_2_ bending at 1427 cm^−1^, for cellulose (crystallized and amorphous), in none of the samples. A slight increase in O-C-O stretching broadening at 1164 cm^−1^, related to cellulose polymerization, was noted in CR, while there was a clear increase in intensity in CH. No changes occurred in O-C-O stretching at 896 cm^−1^, related to crystallized cellulose, in none of the samples. Increased oxidation of cellulose in the treated samples indicates a change in static bending strength.

Figure 7 shows the FTIR spectra of the wood samples of *P. rigida*, where slight changes occurred. The O-H stretching intensity at 3300–3450 cm^−1^ was almost negligible in all three samples. We observed a slight decrease in unconjugated C=O stretching broadening at 1734 cm^−1^, which expressed hemicellulose, in all samples. No changes occurred in conjugated C=O stretching broadening at 1635–1659 cm^−1^, related to cellulose oxidation, in the rusted sample (DR), while there was a clear decrease in intensity in the heated sample (DH), compared with the control (D). We observed a slight decrease in intensity in C=C stretching broadening at 1508 cm^−1^ expressing lignin in all samples. No changes occurred in CH_2_ bending at 1427 cm^−1^, for cellulose (crystallized and amorphous), in none of the samples. We observed a slight decrease in O-C-O stretching broadening at 1164 cm^−1^, related to cellulose polymerization, in DR, while there was a clear decrease in intensity in DH. No changes occurred in O-C-O stretching at 896 cm^−1^, for crystallized cellulose, in DR, while there was a clear decrease in intensity in DH. The decrease in lignin in the treated samples indicates a decrease in the strength of mechanical pressure. An increase in the intensity of the spectra of the functional groups of cellulose in DH indicates an increase in the content of cellulose compared with other chemical compounds.

Figure 8 shows the FTIR spectra of the wood samples of *J. nigra*, where slight changes occurred. The O-H stretching intensity at 3300–3450 cm^−1^ was almost negligible in all three samples. We observed a slight decrease in unconjugated C=O stretching broadening at 1734 cm^−1^, which expressed hemicellulose, in the heated sample (EH), while there was a clear decrease in intensity in the rusted sample (ER), compared with the control (E). No changes occurred in conjugated C=O stretching broadening at 1635–1659 cm^−1^, related to cellulose oxidation, in ER, while there was a slight decrease in intensity in EH, compared with E. A slight decrease in intensity in C=C stretching broadening at 1508 cm^−1^, related to lignin, was found in all samples. No changes occurred in CH_2_ bending at 1427 cm^−1^, related to cellulose (crystallized and amorphous), in EH, while there was a slight decrease in intensity in ER, compared with the control E. No changes occurred in O-C-O stretching broadening at 1164 cm^−1^, related to cellulose polymerization, in ER, while there was a clear increase in intensity in EH. No changes occurred in O-C-O stretching at 896 cm^−1^, related to crystallized cellulose, in none of the samples. The decrease in lignin in the treated samples indicates a decrease in the strength of mechanical pressure.

Wood is an anisotropic material and its decomposition is a complex process. It may be difficult to distinguish and modulate the thermal decomposition behavior of each specific component due to the complexity of wood growth. This causes variance in components’ content, crystal structure and chemical composition from one species to another [81]. Previous FTIR studies showed that lignin is partially oxidized when wood is situated under dry-air conditions during a long period, causing an increase in the relative proportion of carbonyl groups. However, wood under anoxic conditions may undergo hydrolysis followed by leaching of the hydrophilic carbohydrates, contributing to the dominance of the lignin proportion [78,82]. In addition, a high degree of degradation of archeological wood was found, where the average lignin content increased from 25% in fresh wood to up to 45% in archeological wood [83].

The obtained results are very similar to the results of some previous research studies, especially with regard to the effect of thermal ageing and its relationship to the mechanical properties of wood samples [84]. During the heat treatment of wood, cellulose was heavily degraded both in commercial and laboratory heat-treated wood samples, probably due to low pH caused by the heat treatment [85,86]. The decrease in cellulose length in unbuffered systems is of such an extent that it may affect the strength properties of the treated wood [87]. Therefore, the hydrothermal treatment should be performed from neutral to alkaline conditions to avoid cellulose degradation in the wood.

#### 3.1.4. Visual Observation of Inoculated Wood Samples after 7–14 Days

**Figure 9:**
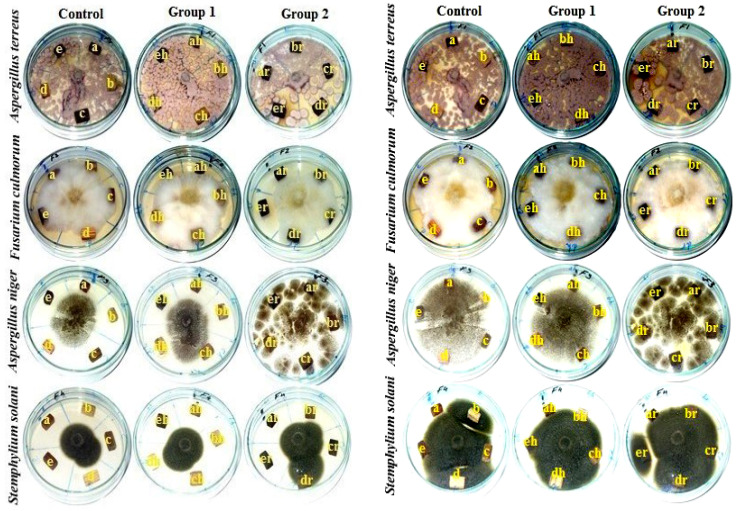
Effects of ageing on five types of wood against four mold fungi.

The effects of ageing on five types of wood against four mold fungi were assessed and compared with the control samples and the visual observations of the growth are presented in Figure 9. No inhibition zones appeared after 14 days of fungal growth of none of the four tested fungi. This is evidence of the lack of influence of accelerated-ageing conditions on the biological resistance of the tested wood samples.

Several studies have discussed the role of metals in general and iron in particular in fungal growth. Iron is one of the most abundant elements on Earth [22,23,27,28,88]. It is required by most living systems and it is an essential element for the growth and development of all living organisms. It is essential to the growth and proliferation of the vast majority of microorganisms. This essentiality derives from the role that iron, in its biochemically accessible valence states, plays in a wide variety of electron transfer processes.

For all fungal pathogens, iron is essential for many metabolic processes and the most intelligent and complex systems of iron acquisition from host cells and tissues is found among various fungal strains [89,90,91]. Iron is highly toxic for biologic substrates, due to its high oxidative potential and its ability to generate reactive oxygen species (ROS) [22]. Fungi can solubilize minerals and metal compounds through several mechanisms, including acidolysis, complexolysis, redoxolysis and by metal accumulation in the biomass. Organic acid excretion by fungi is inter- and intera-specific and can be strongly influenced by the presence of toxic metals [92]. The corrosion reactions can be influenced by microbial activities, especially when the organisms are in close contact with the metal surface, forming a biofilm. The resulting metal deterioration is known as biocorrosion, or microbially influenced corrosion [88].

In wood decay, the cellulose in wood is consumed by organisms until it loses its strength. Wood deterioration can be prevented by impregnation with toxic salts that inhibit fungal growth [93]. Many different terms have been used to describe corrosion caused or induced by microbes, including biocorrosion, microbial corrosion and microbiologically influenced/induced corrosion (MIC). Biocorrosion and microbial corrosion tend to hint that the microbes are the main cause of the corrosion, while MIC suggests an involvement of microbes that may or may not be direct [94].

### 3.2. Pulp Properties

#### 3.2.1. Yield, Kappa Number and Alkali Residue of Pulps

The yield, Kappa number and alkali residue of pulps obtained from the studied wood samples and their treatments are shown in Table 5. *P. rigida* wood showed that the highest pulp yield (%) was obtained from the untreated wood (44.33%), iron-rusted samples (43.56%) and heated samples (41.33%), followed by the untreated wood from *E. humeana* (39.66%), *J. nigra* (39.56%), *T. grandis* (39.46%) and *S. terebinthifolius* (39.4%), while the lowest pulp yield was observed in the heated wood from *T. grandis* (34.63%). In addition, it can be observed that, out of all the woods, the wood samples treated by heating showed a decrease in the pulp yield.

For the Kappa number, the highest number was measured in the pulp of untreated wood from *S. terebinthifolius* (33.66), followed by *P. rigida* (29.66), while the lowest number was measured in the pulp of *T. grandis* heated wood (21.66) and rusted wood (18.33). The residual alkali (g/L) showed the highest concentration in the pulp produced from the untreated woods of *E. humeana* (32.43 g/L), followed by *T. grandis* (28.66 g/L), while the lowest concentrations were observed in the pulps of rusted *S. terebinthifolius* wood (13.50 g/L) and heated wood (15.46) and of the rusted wood of *P. rigida* (15.53 g/L). In addition, it can be noticed that the residual alkali concentrations were decreased in the pulps of heated woods and the lowest values were observed in iron-rusted woods.

#### 3.2.2. Mechanical and Optical Properties of Handsheets

The mechanical and physical properties of the handsheets produced from the studied woods and their treatments (heated and iron-rusted) are shown in Table 6. The highest significant values of tensile strength were observed in pulp paper produced from the untreated, heated and iron-rusted wood of *P. rigida*, with values of 69.66, 65.66 and 68.33 Nm/g, respectively, while the lowest values were found for the handsheets produced from the pulp of the heated woods of *J. nigra* (40.82 Nm/g) and *E. humeana* (40.33 Nm/g) and of *J. nigra* rusted wood (42.56 Nm/g).

We obtained tear resistance values of the handsheets produced from the pulp of woods from untreated *P. rigida* (8.68 mN·m^2^/g), *T. grandis* (7.83 mN·m^2^/g) and rusted *P. rigida* (7.56 mN·m^2^/g); we observed the lowest values in the pulp manufactured from the heated and iron-rusted wood of *E. humeana*, with values of 2.17 and 2.46 mN·m^2^/g, respectively.

The highest burst strength values of the tested paper sheets were reported from the pulp of the untreated woods of *P. rigida* (8.19 kPa·m^2^/g) and *T. grandis* (7.49 kPa·m^2^/g), while the lowest values were observed in the handsheets obtained from *E. humeana* pulps from heated wood (2.14 kPa·m^2^/g) and iron-rusted wood (2.6 kPa·m^2^/g).

The highest fold numbers were reported in the examined handsheets produced from the pulp of the untreated, heated and rusted wood from *P. rigida*, with 195.66, 186.33 and 185.66, respectively, followed by *T. grandis*, with 114.66, 102.33 and 105.33, respectively, while the lowest fold numbers were reported from the pulp produced from untreated, heated and rusted *S. terebinthifolius* woods with values of 9.66, 8.33 and 8.66, respectively.

The highest brightness percentages were found in the handsheets produced from untreated, rusted and heated *J. nigra* wood pulp with values of 38.66, 35.33% and 31.33%, respectively, while the lowest percentages were found in the examined handsheets produced from untreated, heated and rusted wood pulp obtained from *T. grandis* with percentages of 18.33%, 15.33% and 16.33%, respectively.

For the opacity (%), the highest values of 83.66 and 82.66%, were observed in the tested handsheets obtained from the pulp of heated and rusted wood of *T. grandis*, respectively, followed by the rusted wood of *S. terebinthifolius* (76.66%), while the lowest value was found in the handsheets produced from untreated *J. nigra* wood pulp (55.3%). The grammage for all the studied wood materials ranged between 60.13 and 60.23 g/m^2^.

## 4. Conclusions

The findings of the present work confirm the effects of accelerated ageing induced by iron rusting and heating treatments of five wood species on their mechanical, chemical and fungal activity properties. A significant and noticeable decrease was observed in the values of maximum tensile strength parallel to the grain for all aged wood samples compared to the standard samples (untreated). The maximum crushing-strength values were reported in untreated, heated and rusted *T. grandis* wood and the lowest values were observed in the tested untreated, heated and rusted *E. humeana* wood. Through the use of FTIR, we found that the decrease in lignin in the heated sample of *S. terebinthifolius* indicates an expected decrease in mechanical compressive strength, whereas the decrease in lignin in the heated sample of *E. humeana* indicates an expected decrease in mechanical compressive strength. An increase in the intensity of the spectra of the functional groups of cellulose in the heated samples indicates an increase in the content of cellulose, compared to other chemical compounds. According to the biological activity, it was found that no inhibition zones appeared in any of the tested woods against the fungal growth of none of the four tested fungi. The mechanical and optical properties of the handsheets were estimated. The highest significant values of tensile and burst strengths were observed in the pulp paper produced from the untreated, heated and iron-rusted wood of *P. rigida.* Accelerating wood ageing in this study via means of heating and iron rusting resulted in significant differences in the mechanical and chemical properties of wood, as well as the mechanical and physical properties of the produced pulp paper.

## Figures and Tables

**Table 1:** Accelerated ageing steps for wood samples.

Step	Exposure	Temperature (°C)	Time (h)
1	Water soak	49	2
2	Dry-air heat	99	4
3	Water soak	49	2
4	Dry-air heat	99	16

**Table 2:** Effect of heat and rust treatments on the mean values of the selected physical and mechanical properties of the five wood species.

Wood Species	Treatment	MOR (MPa)	MTS (MPa)	C_max_ (MPa)	Density (g/cm^3^)	MC (%)
*Erythrina humeana*	Control	28.91 ± 2 ^H^	20.74 ± 2 ^I^	11.94 ± 1.4 ^G^	0.227 ± 0.04	12.51 ± 0.75
	Heated	28.03 ± 3 ^H^	16.29 ± 4 ^I^	12.12 ± 1.9 ^G^	0.227 ± 0.02	10.31 ± 0.56
	Rusted	23.53 ± 6 ^H^	16.38 ± 4 ^I^	13.54 ± 1.5 ^G^	0.214 ± 0.01	10.11 ± 0.13
*Pinus rigida*	Control	120.46 ± 8 ^C–E^	130.52 ± 4 ^A^	57.82 ± 3.0 ^BC^	0.595 ± 0.02	11.34 ± 0.28
	Heated	139.65 ± 20 ^AB^	60.18 ± 8 ^GH^	52.90 ± 8.2 ^DE^	0.599 ± 0.04	10.53 ± 0.14
	Rusted	124.87 ± 11 ^CD^	89.89 ± 21 ^DE^	53.01 ± 4.9 ^DE^	0.594 ± 0.03	9.91 ± 0.33
*Schinus terebinthifolius*	Control	102.70 ± 4 ^G^	80.22 ± 6 ^EF^	54.45 ± 0.6 ^C–E^	0.538 ± 0.02	11.82 ± 0.67
	Heated	105.96 ± 8 ^F^	64.81 ± 15 ^G^	43.40 ± 6.3 ^F^	0.558 ± 0.04	10.33 ± 0.42
	Rusted	112.05 ± 10 ^E–G^	50.93 ± 5 ^H^	51.99 ± 5.5 ^E^	0.576 ± 0.02	11.93 ± 3.35
*Tectona grandis*	Control	129.18 ± 21 ^BC^	109.85 ± 26 ^B^	65.26 ± 1.3 ^A^	0.588 ± 0.04	10.00 ± 0.46
	Heated	115.79 ± 9 ^D–F^	102.32 ± 12 ^BC^	64.46 ± 3.2 ^A^	0.544 ± 0.02	8.45 ± 0.15
	Rusted	123.70 ± 7 ^CD^	94.24 ± 3 ^CD^	58.75 ± 4.0 ^B^	0.561 ± 0.01	7.34 ± 0.25
*Juglans nigra*	Control	126.10 ± 25 ^CD^	103.17 ± 17 ^BC^	45.09 ± 5.4 ^F^	0.550 ± 0.07	10.07 ± 0.51
	Heated	107.23 ± 12 ^FG^	83.59 ± 22 ^DE^	55.61 ± 5.4 ^B–D^	0.543 ± 0.03	8.67 ± 0.22
	Rusted	144.18 ± 15 ^A^	69.80 ± 10 ^FG^	51.60 ± 5.6 ^E^	0.565 ± 0.08	9.32 ± 0.69
LSD_0.05_		11.67	12.21	3.96	NS	NS

Each value is an average of three samples. C_max_ = the compression strength parallel to grain (MPa) at about 12% moisture content, MTS, maximum tensile strength. Means with the letter/s within the same column are not significantly different according to LSD 0.05.

**Table 3:** Chemical composition of the wood samples.

Wood Species	Treatment	Alcohol:benzene Extractives Content (%)	Lignin Content (%)	Holocellulose Content (%)	Ash Content (%)
*Tectona grandis*	Control	8.23 ± 0.05 ^B^	29.33± 0.57 ^AB^	59.95 ± 0.005 ^N^	2.86 ± 0.01 ^C^
	Heated	7.16 ± 0.005 ^E^	29.66± 0.57 ^A^	61.22 ± 0.005 ^M^	1.96 ± 0.05 ^G^
	Rusted	6.83 ± 0.05 ^F^	29.66 ± 0.57 ^A^	61.34 ± 0.005 ^L^	1.87 ± 0.01 ^H^
*Schinus terebinthifolius*	Control	9.30 ± 0.10 ^A^	24.33 ± 0.57 ^G^	63.76 ± 0.11 ^I^	2.86 ± 0.05 ^C^
	Heated	7.85 ± 0.005 ^C^	25.56 ± 0.05 ^F^	64.56 ± 0.05 ^H^	2.03± 0.01 ^F^
	Rusted	7.65 ± 0.005 ^D^	24.33 ± 0.57 ^G^	65.82± 0.01 ^F^	1.52 ± 0.005 ^I^
*Erythrina humeana*	Control	8.16± 0.01 ^B^	25.66 ± 0.57 ^EF^	61.86± 0.02 ^K^	3.96 ± 0.005 ^A^
	Heated	7.77 ± 0.01 ^CD^	26.66 ± 0.57 ^DE^	62.91± 0.005 ^J^	3.40 ± 0.1 ^B^
	Rusted	5.55 ± 0.005 ^H^	26.33 ± 0.57 ^EF^	64.50 ± 0.10 ^H^	2.85 ±0.005 ^C^
*Pinus rigida*	Control	4.36 ± 0.208 ^J^	28.43 ± 0.11 ^BC^	64.92 ± 0.01 ^G^	2.37 ± 0.01 ^D^
	Heated	3.33 ± 0.02 ^K^	27.66 ± 1.52 ^CD^	66.63 ± 0.208 ^E^	0.85 ± 0.005 ^K^
	Rusted	2.56± 0.05 ^L^	29.60 ± 0.20 ^A^	67.15 ± 0.01 ^D^	0.43± 0.005 ^M^
*Juglans nigra*	Control	6.86 ± 0.005 ^F^	22 ± 1 ^H^	69.97 ± 0.01 ^C^	2.14 ± 0.01 ^E^
	Heated	5.73 ± 0.05 ^G^	22.33 ± 0.57 ^H^	71.25 ± 0.01 ^B^	0.93 ± 0.005 ^J^
	Rusted	5.16 ± 0.11 ^I^	22.60 ± 0.20 ^H^	71.46 ± 0.03 ^A^	0.62 ± 0.01 ^L^
LSD 0.05		0.1224	1.09	0.116	0.057
*p*-value		<0.0001	0.0336	<0.0001	<0.0001

Means with the same letter/s within the same column are not significantly different according to LSD 0.05.

**Table 4:** Basic functional groups and their wavenumbers in natural wood.

Wavenumbers (cm^−1^) *	Functional Group Bands	Assignment
3461	OH stretching	Cellulose, lignin and hemicellulose
2821–2953	CH_2_ stretching	Cellulose, lignin and hemicellulose
1734	Unconjugated C=O stretching	Hemicellulose
1632–1658	Conjugated C=O stretching + H-O-H absorption	Due to oxidation of cellulose
1507	C=C stretching of the aromatic ring	Lignin
1428	CH_2_ bending	Cellulose (crystallized and amorphous)
1164	O-C-O stretching	Cellulose polymerization
1044	C-O stretching	Cellulose, lignin and hemicellulose
898	O-C-O stretching	Crystallized cellulose

* Data according to previously published works [69,70,71,72,73,74,75,76,77,78,79,80].

**Table 5:** Pulp properties from the studied wood samples.

Species	Treatment	Pulp Yield (%)	Kappa Number	Residual Alkali (g/L)
*Tectona grandis*	Control	39.46 ± 0.11 ^DE^	23.33 ± 0.57 ^GH^	28.66 ± 1.52 ^B^
	Heated	34.63 ± 0.05 ^J^	21.66 ± 1.15 ^H^	25.66 ± 0.57 ^D^
	Rusted	36.66 ± 0.57 ^I^	18.33± 0.57 ^I^	19.66 ± 0.57 ^F^
*Schinus terebinthifolius*	Control	39.4 ± 0.1 ^DE^	33.66 ± 0.57 ^A^	18.43 ± 0.11 ^H^
	Heated	36.23 ± 0.11 ^I^	28.33 ± 1.15 ^BC^	15.46± 0.05 ^J^
	Rusted	38.83 ± 0.05 ^EF^	28.33± 0.57 ^BC^	13.50 ± 0.10 ^K^
*Erythrina humeana*	Control	39.66 ± 0.57 ^D^	27.66 ± 1.15 ^CD^	32.43 ± 0.15 ^A^
	Heated	37.56 ± 0.05 ^GH^	26 ± 1 ^DE^	23.46 ± 0.05 ^E^
	Rusted	38.33 ± 1.15 ^F^	25.66 ± 1.52 ^EF^	18.86 ± 0.057 ^F-H^
*Pinus rigida*	Control	44.33 ± 0.57 ^A^	29.66 ± 0.57 ^B^	27.3 ± 0.1 ^C^
	Heated	41.33 ± 0.57 ^C^	28b ± 1 ^C^	18.6 ± 0.2 ^GH^
	Rusted	43.56 ± 0.11 ^B^	26 ± 2 ^DE^	15.53 ± 0.02 ^J^
*Juglans nigra*	Control	39.56 ± 0.15 ^D^	26.66c ± 0.57 ^DE^	27.33 ± 1.15 ^C^
	Heated	37.4 ± 0.10 ^H^	24 ± 2 ^FG^	19.46 ± 0.11 ^FD^
	Rusted	38.16 ± 0.11 ^FG^	22.66 ± 0.57 ^GH^	17 ± 1 ^I^
*p*-value		<0.0001	0.0373	<0.0001

Means with the same letter/s within the same column are not significantly different according to LSD0.05.

**Table 6:** Mechanical and optical properties of handsheets produced from heated or rusted wood pulp compared to untreated control.

Wood species	Treatment	Mechanical Properties				Optical Properties		Grammage (g/m^2^)
		Tensile Strength Nm/g	Tear Resistance mN·m^2^/g	Burst Strength kPa·m^2^/g	Fold Number	Brightness (%)	Opacity (%)	
*Tectona grandis*	Control	57.06 ± 0.66 ^E^	7.83 ± 0.58 ^B^	7.49 ± 0.56 ^B^	114.66 ± 0.57 ^C^	18.33 ± 0.57 ^I^	75.66 ± 0.57 ^B^	60.13 ± 0.05
	Heated	44.56 ± 0.02 ^I^	4.47 ± 0.05 ^F^	3.36 ± 0.02 ^F^	102.33 ± 1.15 ^E^	15.33 ± 0.57 ^J^	83.66 ± 0.57 ^A^	60.16 ± 0.05
	Rusted	52.33 ± 0.57 ^F^	6.23 ± 0.01 ^C^	4.75 ± 0.02 ^E^	105.33 ± 0.57 ^D^	16.33 ± 0.57 ^J^	82.66 ± 0.57 ^A^	60.16 ± 0.05
*Schinus terebinthifolius*	Control	59.66 ± 0.57 ^D^	5.6 ± 0.57 ^D^	4.38 ± 0.57 ^E^	9.66 ± 0.57 ^L^	29.66 ± 0.57 ^D^	65.33 ± 0.57 ^EF^	60.2 ± 0.1
	Heated	50.66 ± 0.57 ^G^	3.25 ± 0.005 ^G^	3.44 ± 0.005 ^F^	8.33 ± 1.15 ^L^	25.33 ± 0.57 ^G^	72.66 ± 0.57 ^C^	60.16 ± 0.05
	Rusted	56.63 ± 0.05 ^E^	3.35 ± 0.01 ^G^	3.76 ± 0.11 ^F^	8.66 ± 0.57 ^L^	28.66 ± 0.57 ^DE^	76.66 ± 1.52 ^B^	60.16 ± 0.11
*Erythrina humeana*	Control	48.66 ± 0.57 ^H^	4.63 ± 0.56 ^F^	4.75 ± 0.01 ^E^	46 ± 1 ^F^	27.33 ± 0.57 ^EF^	62 ± 1.73 ^G^	60.2 ± 0.1
	Heated	40.33 ± 0.57 ^K^	2.17 ± 0.011 ^H^	2.14 ± 0.01 ^H^	38.66 ± 0.57 ^H^	23.33 ± 1.52 ^H^	75.33 ± 1.52 ^BC^	60.20 ± 0.10
	Rusted	44.66 ± 0.57 ^I^	2.46 ± 0.11 ^H^	2.6 ± 0.1 ^G^	42.66 ± 0.57 ^G^	24.33 ± 1.15 ^HG^	68.33 ± 0.57 ^D^	60.20 ± 0.10
*Pinus rigida*	Control	69.66 ± 0.57 ^A^	8.68 ± 0.58 ^A^	8.19 ± 0.58 ^A^	195.66 ± 0.57 ^A^	29.66 ± 0.57 ^D^	67.66 ± 1.52 ^DE^	60.23 ± 0.11
	Heated	65.66 ± 1.52 ^C^	6.64 ± 0.01 ^C^	5.34 ± 0.02 ^D^	186.33 ± 1.52 ^B^	24.66 ± 1.52 ^HG^	75.66 ± 1.52 ^B^	60.23 ± 0.05
	Rusted	68.33 ± 1.15 ^B^	7.56 ± 0.15 ^B^	5.85 ± 0.01 ^D^	185.66 ± 0.57 ^B^	27 ± 1 ^F^	74.66 ± 1.15 ^BC^	60.26 ± 0.05
*Juglans nigra*	Control	45.29 ± 1.16 ^I^	6.54 ± 0.005 ^C^	5.82 ± 0.01 ^D^	24.66 ± 0.57 ^I^	38.66 ± 0.57 ^A^	55.3 ± 0.57 ^H^	60.16 ± 0.05
	Heated	40.82 ± 0.005 ^K^	4.87 ± 0.02 ^EF^	3.55 ± 0.02 ^F^	22 ± 1.73 ^J^	31.33 ± 0.57 ^C^	63 ± 1 ^FG^	60.2 ± 0.1
	Rusted	42.56 ± 0.05 ^J^	5.23 ± 0.01 ^DE^	3.64 ± 0.005 ^F^	20.33 ± 1.52 ^K^	35.33 ± 0.57 ^B^	63 ± 5.19 ^FG^	60.2
*p*-value		<0.0001	<0.0001	<0.0001	<0.0001	0.0028	<0.0001	0.9964

Notes: pulp freeness (csf) of all samples at 300 csf. Means with the same letter/s within the same column are not significantly different according to LSD 0.05.

## Data Availability

Not applicable.
